# CHANGES IN SUBJECTIVE WELL-BEING DURING RETIREMENT TRANSITION: A 10-YEAR COHORT STUDY OF AGING ADULTS IN CHINA

**DOI:** 10.1093/geroni/igac059.1071

**Published:** 2022-12-20

**Authors:** Shuai Zhou

**Affiliations:** The Hong Kong Polytechnic University, Hung Hom, Kowloon, Hong Kong

## Abstract

The literature on retirement adjustment remains inconclusive whether retirement transition is a stressor or a relief. This study examined the effects of retirement on subjective well-being of Chinese ageing adults in various phases of retirement. Drawing on a representative sample of “baby boomers” born in 1945-1965 (N = 3,328) from the China Family Panel Studies (2010-2018), the author examined the within-individual changes in subjective well-being, measured by life satisfaction and confidence in the future, using time-distributed fixed-effects regression technique. After adjusting for a wealth of potential confounders such as financial standing and health, the data reveal that the two outcomes of interest started increasing at two years before retiring. In addition, the improvements persisted in four or more postretirement years. Urban adults experienced greater well-being boost than rural residents during the immediate pre- and post-retirement period, but not in two or more postretirement years. Furthermore, the subjective well-being of urban females and rural males increased upon retirement and fluctuated significantly, whereas rural females and urban males experienced a stable increase in subjective well-being since the first preretirement year. Results suggest that the transition into retirement generally represents a relief for urban ageing adults in China, but the well-being improvements are stratified by residency and gender. Therefore, retirement policies should ensure subjective well-being of disadvantaged pre-retirees and attend to the needs of retired urban women and rural men.

